# Modeling the survival times of the COVID-19 patients with a new statistical model: A case study from China

**DOI:** 10.1371/journal.pone.0254999

**Published:** 2021-07-26

**Authors:** Xiaofeng Liu, Zubair Ahmad, Ahmed M. Gemeay, Alanazi Talal Abdulrahman, E. H. Hafez, N. Khalil

**Affiliations:** 1 Shanghai University of Finance and Economics Zhejiang College, Jinhua City, Zhejiang Province, China; 2 Department of Statistics, Yazd University, Yazd, Iran; 3 Department of Mathematics, Faculty of Science, Tanta University, Tanta, Egypt; 4 Department of Mathematics, College of Science University of Ha’il, Hail, Saudi Arabia; 5 Department of Mathematics, Faculty of Science, Helwan University, Cairo, Egypt; 6 Department of Mathematics, College of Science and Arts, Qassim University, Ar Rass, Saudi Arabia; Tongii University, CHINA

## Abstract

Over the past few months, the spread of the current COVID-19 epidemic has caused tremendous damage worldwide, and unstable many countries economically. Detailed scientific analysis of this event is currently underway to come. However, it is very important to have the right facts and figures to take all possible actions that are needed to avoid COVID-19. In the practice and application of big data sciences, it is always of interest to provide the best description of the data under consideration. The recent studies have shown the potential of statistical distributions in modeling data in applied sciences, especially in medical science. In this article, we continue to carry this area of research, and introduce a new statistical model called the arcsine modified Weibull distribution. The proposed model is introduced using the modified Weibull distribution with the arcsine-*X* approach which is based on the trigonometric strategy. The maximum likelihood estimators of the parameters of the new model are obtained and the performance these estimators are assessed by conducting a Monte Carlo simulation study. Finally, the effectiveness and utility of the arcsine modified Weibull distribution are demonstrated by modeling COVID-19 patients data. The data set represents the survival times of fifty-three patients taken from a hospital in China. The practical application shows that the proposed model out-classed the competitive models and can be chosen as a good candidate distribution for modeling COVID-19, and other related data sets.

## 1 Introduction

The first outbreak of the current COVID-19 epidemic was first seen in the popular seafood market in the Chinese city of Wuhan, where large numbers of people come to buy or sell seafood. As of December 31, 2019, a total of 27 cases of COVD-19 epidemic were reported by the WMHC (Wuhan Municipal Health Commission). After highly effecting some Chines cities and provinces, this pandemic transmitted to other countries via air routes [1].

Almost every country around the globe has paid a huge price in terms of financial and human loss, and yet, some countries are paying and in sacrifice process. The countries having large population densities and low health facilities have higher chances of being in critical situations during this pandemic [2].

**Fig 1:**
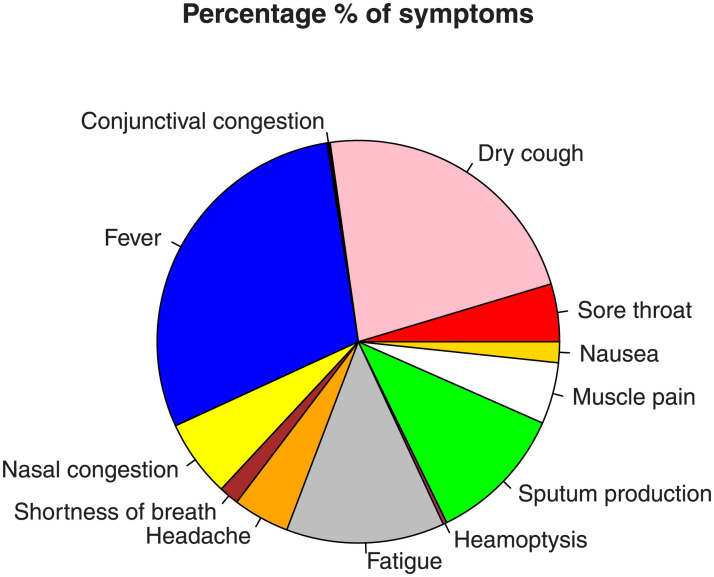
Graphical display for the percentage of the symptoms of COVID-19.

The main serious and common symptoms attributed to COVID-19 are sore throat (13.9%), dry cough (67.7%), Fever (87.9%), shortness of breath (18.6%), headache (13.6%), fatigue (38.1%), sputum production (33.4%), muscle pain (14.8%), nausea (5.0%), nasal congestion (4.8%), haemoptysis (0.9%) and conjunctival congestion (0.8%). For more detail about the symptoms of COVID-19 pandemic; see [3]. The % of symptoms are displayed in Fig 1.

The comparison of COVID-19 epidemic between different countries is worth of studies and is of great concern. In this regard, researchers are devoting great efforts to make comparisons between different countries. For some previous attempts to compare this epidemic in Italy and China; see [4] for details. Comparison of COVID-19 in Europe, USA, and South Korea is provided in [5]. The COVID-19 pandemic in Australia has been discussed in [6].

Modeling the spread of COVID-19 in Lebanon is provided in [7]. A case study form Spain has been studied in [8]. Mathematical analysis of COVID-19 in Mexico is provided in [9]. A case study form Brazil is discussed in [10]. The progress of COVID-19 epidemic in Pakistan is studied by [11]. A mathematical model for COVID-19 transmission dynamics in India is provided by [12]. Al-Babtain et al. [13] introduced two case studies in Saudi Arabia, the first one about COVID-19 infections from 24 March to 12 April, 2020 and the second about numbers of daily recover patients in the same period of time. Comparison of COVID-19 events in Asian countries has been carried out in [14]. The comparison between Iran and mainland China has appeared in [15]. The comparison between the two neighbour countries Iran and Pakistan has appeared in [16]. A case of the COVID-19 pandemic in Indonesia has been discussed in [17]. For more information, reader can refer to [18–27].

In the current situation, it is of great interest to study more about COVID-19 to make comparison between different countries. In the domain and practice of big data science, to provide the best description of the data under consideration is a prominent research topic. The recent studies have pointed out the applicability of statistical models to provide the best description of the random phenomena. In this article, we focus on this research area of distribution theory, and introduce a new statistical model to provide the best fit to data in linked with COVID-19 and other related events.

The modified Weibull distribution is one of the most prominent modifications of the Weibull distribution which is introduced to improve the fitting power of the exponential, Rayleigh, linear failure rate and Weibull distributions; see [28]. We further carry this area of distribution theory and introduce a new prominent version of the modified Weibull distribution to improve its fitting power. A random variable *X*, is said to follow the modified Weibull distribution with shape parameter *α* and scale parameters *κ*_1_ and *κ*_2_, if its cdf (cumulative distribution function) denoted *F*(*x*;Ξ), is given by

$$\begin{array}{c} F\left(x;\Xi \right)=1-e^{-\kappa_{1}x^{\alpha }-\kappa_{2}x},\;\;\;\;\;\;\;\;\;\;x\geq 0,\alpha ,\kappa_{1},\kappa_{2}>0, \end{array}$$ (1)

where Ξ = (*α*, *κ*_1_, *κ*_2_). The pdf (probability density function) corresponding to expression Eq 1 is

$$\begin{array}{c} f\left(x;\Xi \right)=\left(\alpha \kappa_{1}x^{\alpha -1}+\kappa_{2}\right)e^{-\kappa_{1}x^{\alpha }-\kappa_{2}x},\;\;\;\;\;\;\;\;\;\;x>0. \end{array}$$

In this article, we focus on proposing a new modification of the modified Weibull distribution called the arcsine modified Weibull (ASM-Weibull) distribution. The ASM-Weibull distribution is introduced by adopting the approach of the arcsine-*X* distributions of [29], which can be obtained as a sub-case of [30]. The cdf and pdf of the arcsine-*X* distributions are given, respectively, by

$$\begin{array}{c} G\left(x\right)=\frac{2}{\pi }arcsine\left(F\left(x;\Xi \right)\right),\;\;\;\;\;\;\;\;\;\;x\in R. \end{array}$$ (2)

where *F*(*x*;Ξ) is cdf of the baseline random variable. The respective pdf is

$$\begin{array}{c} g\left(x\right)=\frac{2}{\pi }\frac{f(x;\Xi )}{\sqrt{1-F{\left(x;\Xi \right)}^{2}}},\;\;\;\;\;\;\;\;\;\;x\in R. \end{array}$$

The cdf of the proposed ASM-Weibull distribution is obtained by using the expression 1 in 2. The flexibility and applicability of the ASM-Weibull distribution are examined via an application to the survival times of the COVID-19 patient data.

## 2 The arcsine-modified weibull model

In this section, we introduce the ASM-Weibull distribution. A random variable *X*, is said to follow the ASM-Weibull distribution, if its cdf is given by

$$\begin{array}{c} G\left(x\right)=\frac{2}{\pi }arcsine\left(1-e^{-\kappa_{1}x^{\alpha }-\kappa_{2}x}\right),\;\;\;\;\;\;\;\;\;\;x\geq 0,\alpha ,\kappa_{1},\kappa_{2}>0. \end{array}$$ (3)

The density function corresponding to 3 is given by

$$\begin{array}{c} g\left(x\right)=\frac{2}{\pi }\frac{\left(\alpha \kappa_{1}x^{\alpha -1}+\kappa_{2}\right)e^{-\kappa_{1}x^{\alpha }-\kappa_{2}x}}{\sqrt{1-{\left(1-e^{-\kappa_{1}x^{\alpha }-\kappa_{2}x}\right)}^{2}}},\;\;\;\;\;\;\;\;\;\;x>0. \end{array}$$ (4)

**Fig 2:**
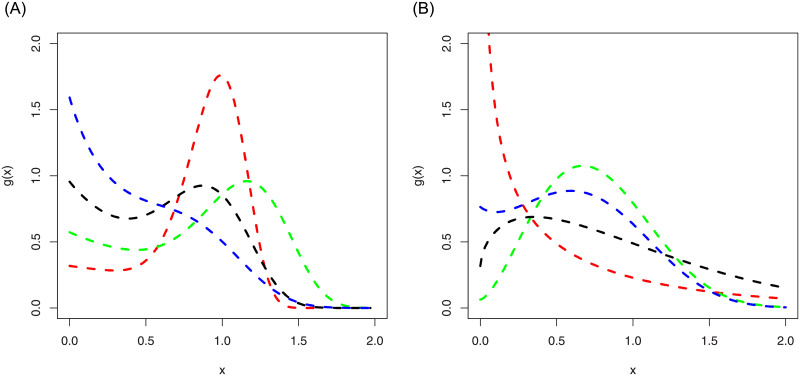
Different density plots of the ASM-Weibull distribution.

Some possible behaviors of the pdf of the ASM-Weibull distribution are shown in Fig 2. The plots in the right panel of Fig 2, are sketched for *α* = 6.5, *κ*_1_ = 1.5, *κ*_2_ = 0.5 (red-line), *α* = 5.4, *κ*_1_ = 0.5, *κ*_2_ = 0.9 (green-line), *α* = 4.6, *κ*_1_ = 1.5, *κ*_2_ = 1.5 (black-line), *α* = 3.8, *κ*_1_ = 1.5, *κ*_2_ = 2.5 (blue-line). Whereas, the plots in the left panel of Fig 2, are presented for *α* = 0.5, *κ*_1_ = 1.5, *κ*_2_ = 1.5 (red-line), *α* = 2.5, *κ*_1_ = 2.5, *κ*_2_ = 0.1 (green-line), *α* = 1.6, *κ*_1_ = 1.2, *κ*_2_ = 0.5 (black-line), *α* = 2.8, *κ*_1_ = 1.8, *κ*_2_ = 1.2 (blue-line).

**Fig 3:**
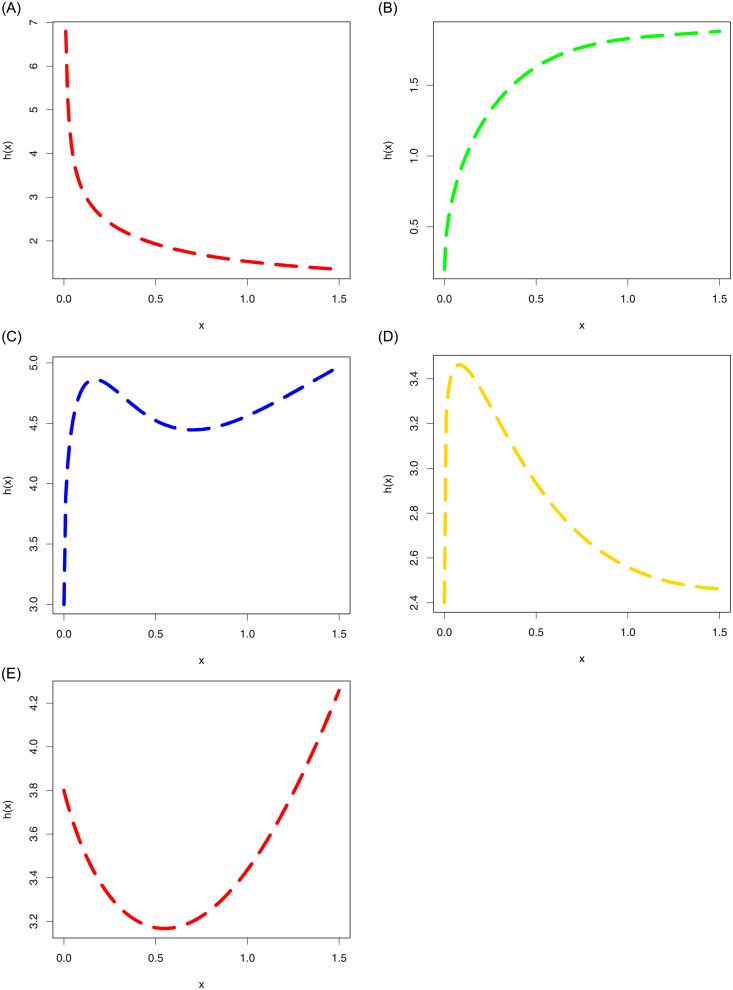
Different hrf plots of the ASM-Weibull distribution.

Some possible behaviors of the hazard rate function (hrf) *h*(*x*) of the ASM-Weibull distribution are shown in Fig 3. The plots provided in Fig 3, are sketched for *α* = 0.5, *κ*_1_ = 0.5, *κ*_2_ = 1 (red-line), *α* = 1.5, *κ*_1_ = 0.8, *κ*_2_ = 0.5 (green-line), *α* = 1.5, *κ*_1_ = 2.1, *κ*_2_ = 1.5 (blue-line),*α* = 1.2, *κ*_1_ = 0.9, *κ*_2_ = 1.2 (gold-line), *α* = 2.4, *κ*_1_ = 0.5, *κ*_2_ = 1.8 (black-line). From the plots provided in Fig 3, we can see that the proposed model captures different important behaviours of the hrf such as increasing, decreasing, unimodal also called upside down bathtub, modified unimodal and most importantly bathtub shapes.

## 3 Basic mathematical properties

This section deals with the computation of some statistical properties of the ASM-Weibull distribution.

### 3.1 Quantile function

Let *X* denote the ASM-Weibull random variable with cdf 3, then the qf (quantile function) of *X*, denoted *Q(u)*, is given by

$$\begin{array}{c} Q\left(u\right)=\kappa_{1}x^{\alpha }+\kappa_{2}x+\log(1-sin(\frac{\pi }{2}u)). \end{array}$$ (5)

where *u* has the uniform distribution on the interval (0,1).

### 3.2 Moments

This subsection deals with the computation of *r*^*th*^ moment of the ASM-Weibull distribution that can be further used to obtain important characteristics. It is often employed in computing the main properties and characteristics of the distribution (as an example of this characteristics skewness, central tendency, dispersion, and kurtosis). In this section, we derive the *r*^*th*^ moment of the ASM-Weibull distribution as follows

$$\begin{array}{c} \mu_{r}^{\prime }=\frac{2}{\pi }\int_{0}^{\infty }x^{r}\frac{\left(\alpha \kappa_{1}x^{\alpha -1}+\kappa_{2}\right)e^{-\kappa_{1}x^{\alpha }-\kappa_{2}x}}{\sqrt{1-{\left(1-e^{-\kappa_{1}x^{\alpha }-\kappa_{2}x}\right)}^{2}}}dx. \end{array}$$ (6)

Using binomial series that is convergent when |*t*| < 1 (see, https://socratic.org/questions/how-do-you-use-the-binomial-series-to-expand-f-x-1-sqrt-1-x-2), we have

$$\begin{array}{c} \frac{1}{\sqrt{1-t^{2}}}=\sum_{n=0}^{\infty }\frac{1\times 3\times 5\times \ldots \times (2n-1)}{n!2^{n}}t^{2n}. \end{array}$$ (7)

Using 7, we have

$$\begin{array}{c} \frac{1}{\sqrt{1-{\left(1-e^{-\kappa_{1}x^{\alpha }-\kappa_{2}x}\right)}^{2}}}=\sum_{n=0}^{\infty }\frac{1\times 3\times 5\times \ldots \times (2n-1)}{n!2^{n}}{\left(1-e^{-\kappa_{1}x^{\alpha }-\kappa_{2}x}\right)}^{2n}. \end{array}$$ (8)

The expression 8 can also be written as

$$\begin{array}{cc} \frac{1}{\sqrt{1-{\left(1-e^{-\kappa_{1}x^{\alpha }-\kappa_{2}x}\right)}^{2}}} & =\sum_{n=0}^{\infty }\sum_{i=0}^{2n}\left(\begin{array}{c} 2n\\ i \end{array}\right){\left(-1\right)}^{i}\frac{1\times 3\times 5\times \ldots \times (2n-1)}{n!2^{n}}\\ \; & \times e^{-i\kappa_{1}x^{\alpha }-i\kappa_{2}x}. \end{array}$$ (9)

Using expression 9 in 6, we have

$$\begin{array}{cc} \mu_{r}^{\prime } & =\frac{2}{\pi }\sum_{n=0}^{\infty }\sum_{i=0}^{2n}\left(\begin{array}{c} 2n\\ i \end{array}\right){\left(-1\right)}^{i}\frac{1\times 3\times 5\times \ldots \times (2n-1)}{n!2^{n}}\\ & \times \int_{0}^{\infty }x^{r}\left(\alpha \kappa_{1}x^{\alpha -1}+\kappa_{2}\right)e^{-\kappa_{1}x^{\alpha }\left(i+1\right)-\kappa_{2}x\left(i+1\right)}dx. \end{array}$$ (10)

Using the series *e*^−*t*^, we have

$$\begin{array}{c} e^{-t}=\sum_{j=0}^{\infty }\frac{{\left(-1\right)}^{j}}{j!}t^{j}. \end{array}$$ (11)

Let *t* = *κ*_1_(*i* + 1)*x*^*α*^, then using the expression 11, we get

$$\begin{array}{c} e^{-\kappa_{1}\left(i+1\right)x^{\alpha }}=\sum_{j=0}^{\infty }\frac{{\left(-1\right)}^{j}{\left(i+1\right)}^{j}\kappa_{1}^{j}}{j!}x^{j\alpha }. \end{array}$$ (12)

Using expression 12 in 10, we get

$$\begin{array}{cc} \mu_{r}^{\prime } & =\frac{2}{\pi }\sum_{n=0}^{\infty }\sum_{i=0}^{2n}\left(\begin{array}{c} 2n\\ i \end{array}\right){\left(-1\right)}^{i+j}{\left(i+1\right)}^{j}\kappa_{1}^{j}\frac{1\times 3\times 5\times \ldots \times (2n-1)}{n!2^{n}}\\ & \times \int_{0}^{\infty }x^{r+j\alpha }\left(\alpha \kappa_{1}x^{\alpha -1}+\kappa_{2}\right)e^{-\kappa_{2}x\left(i+1\right)}dx, \end{array}$$

$$\begin{array}{cc} \mu_{r}^{\prime } & =\frac{2}{\pi }\sum_{n=0}^{\infty }\sum_{i=0}^{2n}\left(\begin{array}{c} 2n\\ i \end{array}\right){\left(-1\right)}^{i+j}{\left(i+1\right)}^{j}\kappa_{1}^{j}\frac{1\times 3\times 5\times \ldots \times (2n-1)}{n!2^{n}}\\ & \times [\alpha \kappa_{1}\int_{0}^{\infty }x^{r+\alpha (j+1)-1}e^{-\kappa_{2}\left(i+1\right)x}dx+\kappa_{2}\int_{0}^{\infty }x^{r+j\alpha }e^{-\kappa_{2}\left(i+1\right)x}dx], \end{array}$$

$$\begin{array}{cc} \mu_{r}^{\prime } & =\frac{2}{\pi }\sum_{n=0}^{\infty }\sum_{i=0}^{2n}\left(\begin{array}{c} 2n\\ i \end{array}\right){\left(-1\right)}^{i+j}{\left(i+1\right)}^{j}\kappa_{1}^{j}\frac{1\times 3\times 5\times \ldots \times (2n-1)}{n!2^{n}}\\ & \times [\alpha \kappa_{1}\frac{\Gamma (r+\alpha (j+1))}{{\left(\kappa_{2}\left(i+1\right)\right)}^{r+\alpha (j+1)}}+\kappa_{2}\frac{\Gamma (r+j\alpha +1)}{{\left(\kappa_{2}\left(i+1\right)\right)}^{r+j\alpha +1}}]. \end{array}$$

**Fig 4:**
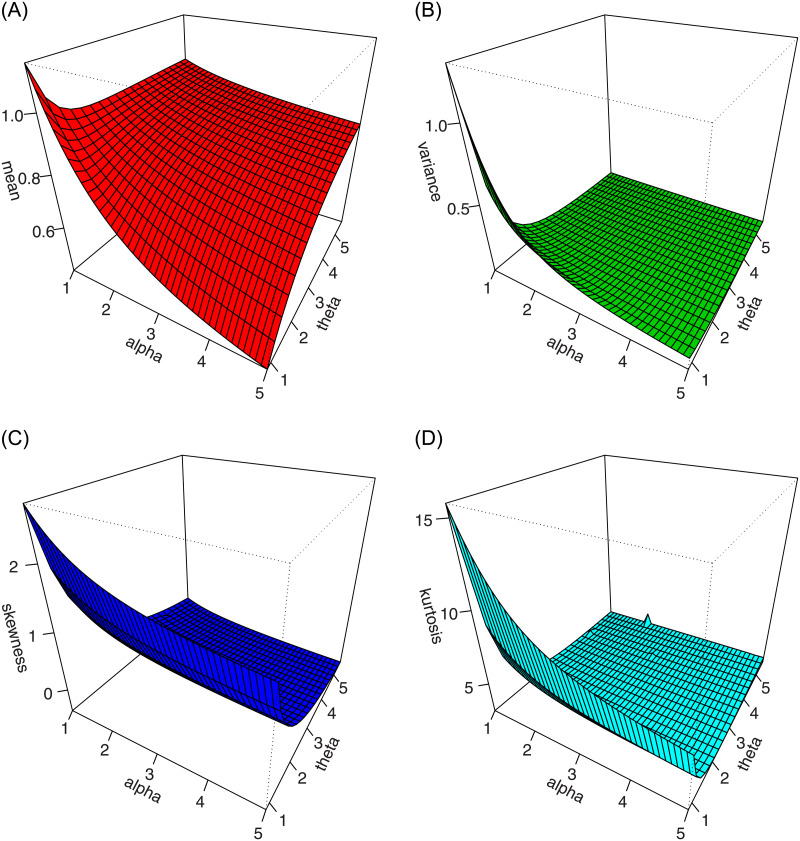
Plots of the mean, variance, skewness and kurtosis for *κ*_1_ = 1.2 and different values of *α* and *κ*_2_.

**Fig 5:**
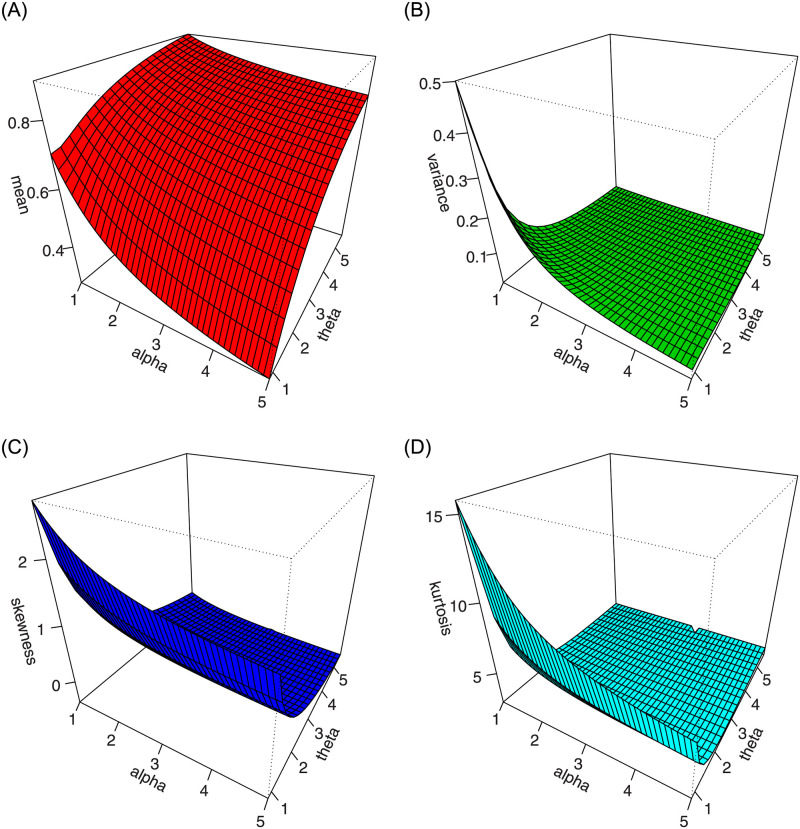
Plots of the mean, variance, skewness and kurtosis for *κ*_1_ = 0.5 and different values of *α* and *κ*_2_.

For different values of *α* and *κ*_2_, and fixed value of *κ*_1_, the plots of mean, variance, skewness, and kurtosis of the ASM-Weibull distribution are presented in Figs 4 and 5.

Furthermore, the mgf (moment generating function) of the ASM-Weibull distribution denoted by *M*_*X*_(*t*) has the form

$$\begin{array}{c} M_{X}\left(t\right)=\frac{2}{\pi }\sum_{r,n=0}^{\infty }\sum_{i=0}^{2n}\left(\begin{array}{c} 2n\\ i \end{array}\right){\left(-1\right)}^{i}\frac{1\times 3\times 5\times \ldots \times (2n-1)t^{r}}{r!n!2^{n}}\eta_{r,i,\alpha ,\kappa_{1},\kappa_{2}}. \end{array}$$

## 4 Maximum likelihood estimation

Here, we derive the maximum likelihood estimators (MLEs) of the ASM-Weibull distribution parameters based on the complete samples only. Let *x*_1_, *x*_2_, …, *x*_*n*_ represent the observed values from the ASM-Weibull distribution with parameters *α*, *κ*_1_ and *κ*_2_. Corresponding to Eq 4, the total log-likelihood function ℓ(*α*, *κ*_1_, *κ*_2_) is given by

$$\begin{array}{cc} \ell (\alpha ,\kappa_{1},\kappa_{2}) & =n\log(\frac{2}{\pi })+\sum_{i=1}^{n}\log(\alpha \kappa_{1}x_{i}^{\alpha -1}+\kappa_{2})-\sum_{i=1}^{n}\kappa_{1}x_{i}^{\alpha }-\sum_{i=1}^{n}\kappa_{2}x_{i}\\ & -\frac{1}{2}\sum_{i=1}^{n}(1-{\left(1-e^{-\kappa_{1}x_{i}^{\alpha }-\kappa_{2}x_{i}}\right)}^{2}). \end{array}$$ (13)

The numerical maximization of ℓ(*α*, *κ*_1_, *κ*_2_) can be done either by using the computer software or via differentiation on behalf of *α*, *κ*_1_ and *κ*_2_. Corresponding to Eq 13, the partial derivatives are as follows

$$\begin{array}{cc} \frac{\partial }{\partial \alpha }\ell \left(\alpha ,\kappa_{1},\kappa_{2}\right) & =\kappa_{1}\sum_{i=1}^{n}\frac{x_{i}^{\alpha }(\log x_{i})e^{-\kappa_{1}x_{i}^{\alpha }}\left(1-e^{-\kappa_{1}x_{i}^{\alpha }-\kappa_{2}x_{i}}\right)}{\left(1-{\left(1-e^{-\kappa_{1}x_{i}^{\alpha }-\kappa_{2}x_{i}}\right)}^{2}\right)}-\kappa_{1}\sum_{i=1}^{n}x_{i}^{\alpha }(\log x_{i})\\ & +\kappa_{1}\sum_{i=1}^{n}\frac{x_{i}^{\alpha -1}[\alpha (\log x_{i})+1]}{\left(\alpha \kappa_{1}x^{\alpha -1}+\kappa_{2}\right)}, \end{array}$$ (14)

$$\begin{array}{c} \frac{\partial }{\partial \kappa_{1}}\ell \left(\alpha ,\kappa_{1},\kappa_{2}\right)=\sum_{i=1}^{n}\frac{x_{i}^{\alpha }e^{-\kappa_{1}x_{i}^{\alpha }}\left(1-e^{-\kappa_{1}x_{i}^{\alpha }-\kappa_{2}x_{i}}\right)}{\left(1-{\left(1-e^{-\kappa_{1}x_{i}^{\alpha }-\kappa_{2}x_{i}}\right)}^{2}\right)}-\sum_{i=1}^{n}x_{i}^{\alpha }+\sum_{i=1}^{n}\frac{\alpha x_{i}^{\alpha -1}}{\left(\alpha \kappa_{1}x^{\alpha -1}+\kappa_{2}\right)}, \end{array}$$ (15)

$$\begin{array}{c} \frac{\partial }{\partial \kappa_{2}}\ell \left(\alpha ,\kappa_{1},\kappa_{2}\right)=\sum_{i=1}^{n}\frac{x_{i}e^{-\kappa_{2}x_{i}}\left(1-e^{-\kappa_{1}x_{i}^{\alpha }-\kappa_{2}x_{i}}\right)}{\left(1-{\left(1-e^{-\kappa_{1}x_{i}^{\alpha }-\kappa_{2}x_{i}}\right)}^{2}\right)}-\sum_{i=1}^{n}x_{i}+\sum_{i=1}^{n}\frac{1}{\left(\alpha \kappa_{1}x^{\alpha -1}+\kappa_{2}\right)}. \end{array}$$ (16)

Solving $\frac{\partial }{\partial \alpha }\ell \left(\alpha ,\kappa_{1},\kappa_{2}\right)=\frac{\partial }{\partial \kappa_{1}}\ell \left(\alpha ,\kappa_{1},\kappa_{2}\right)=\frac{\partial }{\partial \kappa_{2}}\ell \left(\alpha ,\kappa_{1},\kappa_{2}\right)=0$, yield the MLEs $\left(\hat{\alpha },\hat{\kappa_{1}},\hat{\kappa_{2}}\right)$ of (*α*, *κ*_1_, *κ*_2_). For more information and extensive reading about MLEs, we refer to [31–34].

## 5 Simulation study

In this section of the paper, we provide a brief Monte Carlo simulation study to evaluate the MLEs of the ASM-Weibull distribution parameters. The ASM-Weibull distribution is easily simulated by inverting the expression 3. Let *U* has a uniform distribution *U*(0,1), then the nonlinear equation by inverting Eq 3 is

$$\begin{array}{c} \kappa_{1}x^{\alpha }+\kappa_{2}x+\log(1-sin(\frac{\pi }{2}u)). \end{array}$$ (17)

The simulation is performed for two different sets of parameters (i) *α* = 0.7, *κ*_1_ = 1, *κ*_2_ = 0.5, and (ii) *α* = 1.2, *κ*_1_ = 1.4, *κ*_2_ = 0.5.

The random number generation is obtained via the inverse cdf. The inverse process and simulation results are obtained via a statistical software R using (rootSolve) library with command mle. The sample size selected as *n* = 10, 20, …, 500 and the Monte Carlo replications made was 500 times. For the maximization of the expression 13, the algorithm “LBFGS-B” is used with optim(). For *i* = 1, 2, …, 500, the MLEs $\left(\hat{\alpha },\hat{\kappa_{1}},\hat{\kappa_{2}}\right)$ of (*α*, *κ*_1_, *κ*_2_) are obtained for each set of simulated data. The assessing tools such as biases and mean square errors (MSEs) are considered. These quantities are calculated as follows

$$\begin{array}{c} Bias(\Theta )=\frac{1}{500}\sum_{i=1}^{500}(\hat{\Theta }-\Theta ), \end{array}$$

and

$$\begin{array}{c} MSE(\Theta )=\frac{1}{500}\sum_{i=1}^{500}{\left(\hat{\Theta }-\Theta \right)}^{2}, \end{array}$$

where Θ = (*α*, *κ*_1_, *κ*_2_). The coverage probabilities (CPs) are calculated at the 95% confidence interval (C.I).

**Fig 6:**
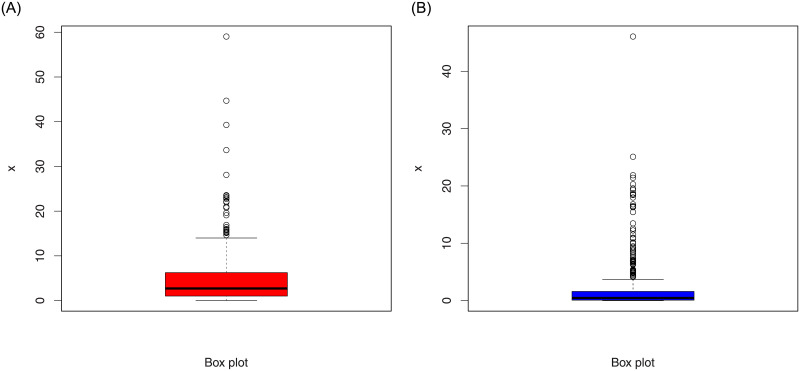
Box plots of the simulated data set 1 (red color) and set 2 (blue color).

The summary measures of the simulated data presented in Table 1 and the box plots are provided in Fig 6.

**Table 1:** The summary measures of the simulated data sets.

Simulated Data	Min.	1st Qu.	Median	Mean	3rd Qu.	Max.
Set 1	0.001	1.013	2.710	4.736	6.232	59.042
Set 2	0.0000	0.0738	0.4550	1.9608	1.5947	46.0600

**Fig 7:**
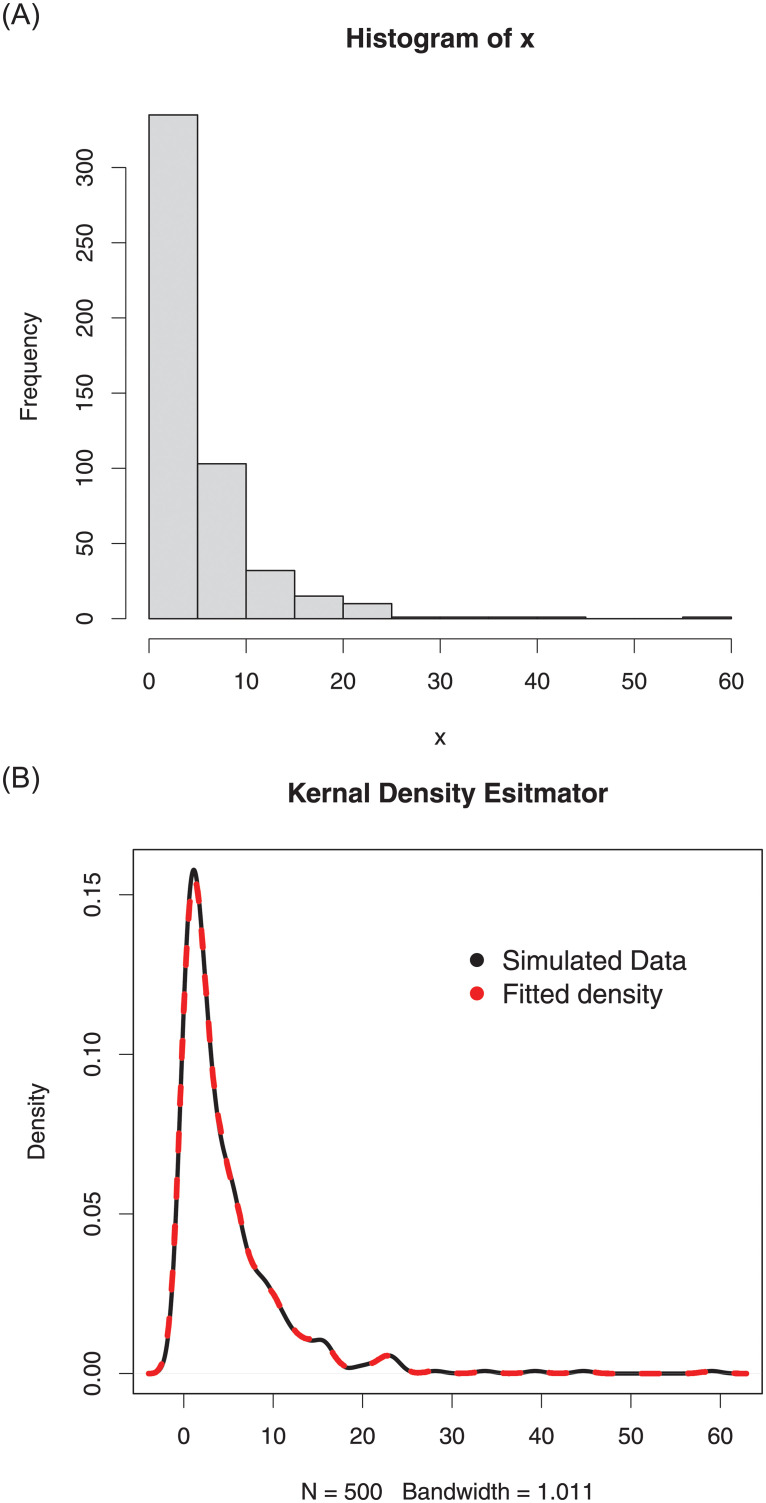
The histogram and Kernel density estimator of the simulated data set 1.

**Fig 8:**
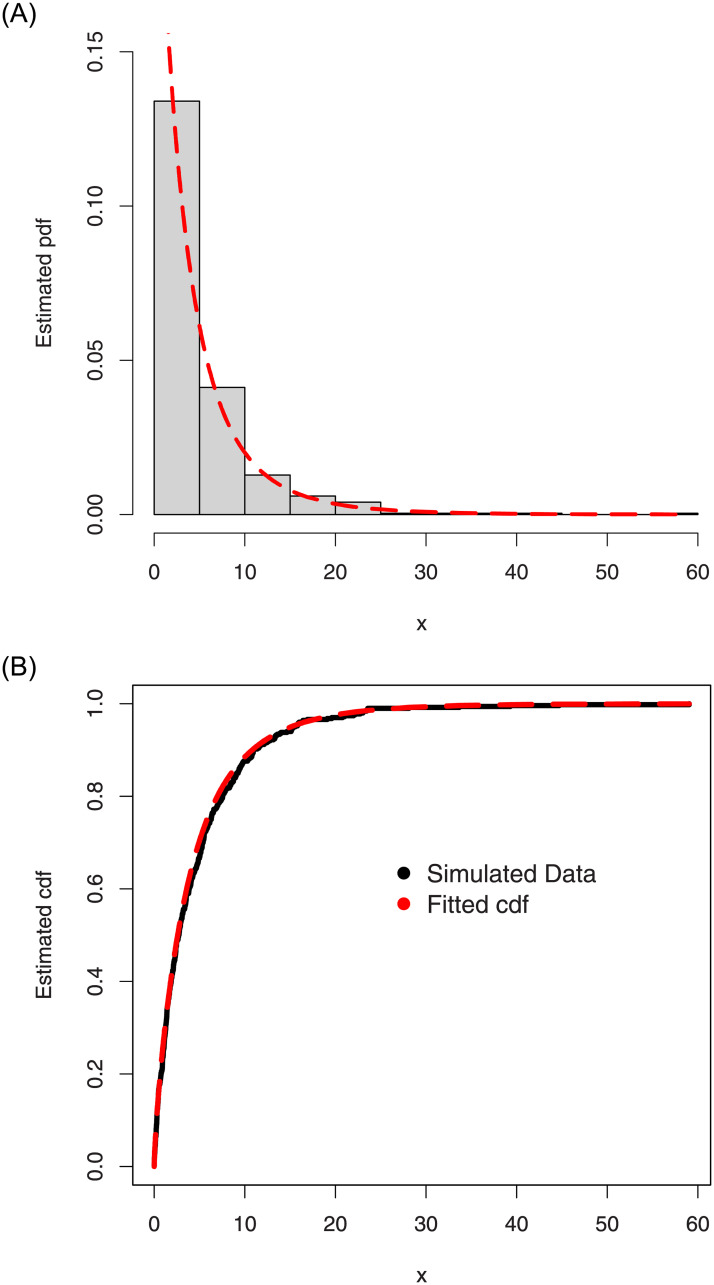
The fitted pdf and cdf of the ASM-Weibull model for the simulated data set 1.

**Fig 9:**
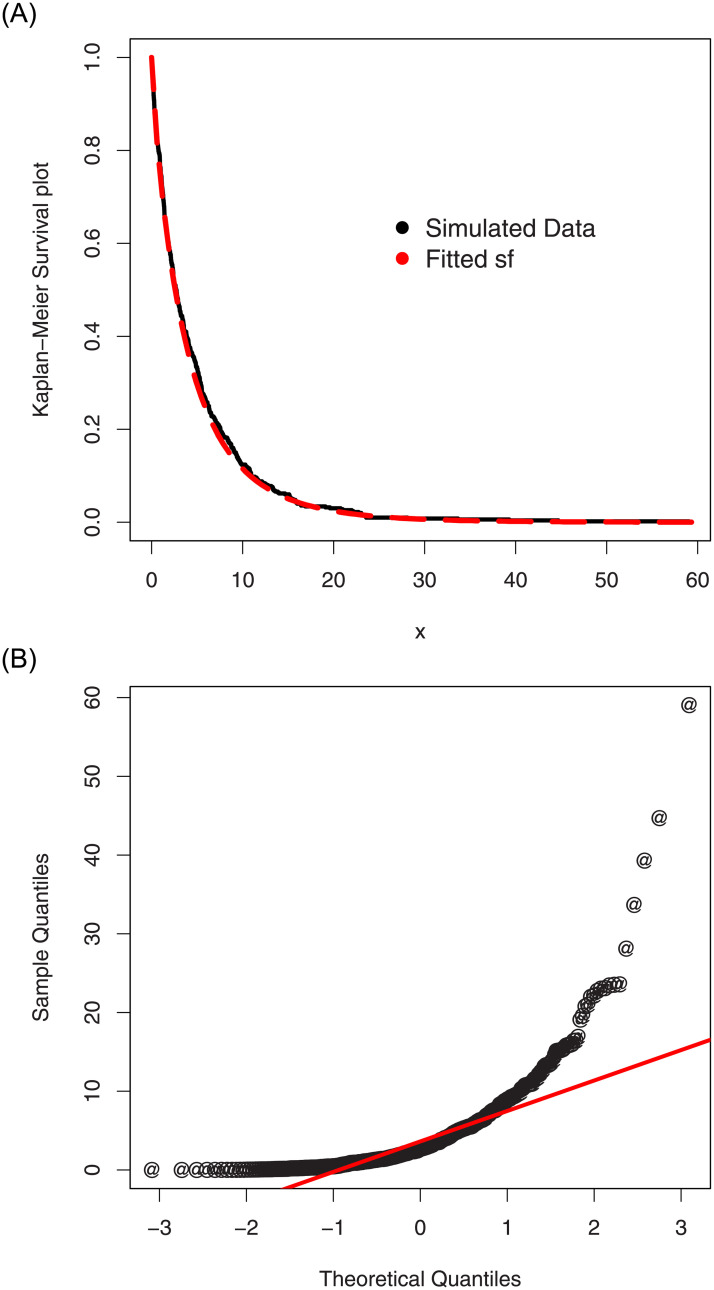
The QQ and Kaplan-Meier survival plots of the ASM-Weibull model for the simulated data set 1.

For the simulated data set 1, (i) the histogram and Kernel density estimator are presented in Fig 7, (ii) the fitted pdf and cdf are sketched in Fig 8, and (iii) the Kaplan-Meier survival and QQ (quantile-quantile) plots are provided in Fig 9. Corresponding to the first set of parameters values, the simulations results are provided in Table 2.

**Table 2:** The Monte Carlo simulation results of the ASM-Weibull distribution.

	Set 1: *α* = 0.7, *κ*_1_ = 1, *κ*_2_ = 0.5						
*n*	Par.	MLE	MSEs	Biases	Confidence Interval	CPs	Variances
10	*α*	0.95163	0.30983	0.24163	(0.30356, 1.47970)	0.982	0.90602
	*κ*_1_	1.41624	2.97415	0.41624	(-4.62888, 3.46137)	0.990	1.56130
	*κ*_2_	0.81004	0.41880	0.21004	(-1.73901, 3.15911)	0.884	2.92010
20	*α*	0.90693	0.27431	0.20693	(0.40446, 1.20940)	0.978	0.84216
	*κ*_1_	1.39378	2.18688	0.35937	(-2.39180, 3.19793)	0.976	1.20960
	*κ*_2_	0.79625	0.37974	0.19625	(-1.41940, 2.89190)	0.890	2.71498
100	*α*	0.86174	0.21262	0.14174	(0.54005, 0.94342)	0.906	0.61058
	*κ*_1_	1.36827	1.41030	0.29827	(-1.11438, 3.06107)	0.956	0.98438
	*κ*_2_	0.73479	0.29131	0.15479	(-0.41042, 1.68002)	0.912	1.45309
200	*α*	0.83339	0.15829	0.10339	(0.56393, 0.90399)	0.926	0.47525
	*κ*_1_	1.32276	0.60629	0.16276	(-0.41898, 2.54452)	0.950	0.17330
	*κ*_2_	0.63017	0.14327	0.13017	(-0.18576, 1.44611)	0.908	0.57153
300	*α*	0.79676	0.10642	0.09676	(0.58486, 0.86866)	0.903	0.15241
	*κ*_1_	1.21962	0.39405	0.10962	(-0.15800, 2.19726)	0.924	0.11109
	*κ*_2_	0.60906	0.11851	0.10906	(-0.04422, 1.26235)	0.958	0.36099
400	*α*	0.76610	0.07600	0.05610	(0.59241, 0.83979)	0.924	0.03982
	*κ*_1_	1.15222	0.29089	0.09222	(0.04500, 2.17945)	0.934	0.07579
	*κ*_2_	0.57035	0.09127	0.07035	(0.03073, 1.10997)	0.948	0.29648
500	*α*	0.71224	0.00547	0.02224	(0.60464, 0.83984)	0.940	0.00359
	*κ*_1_	1.08212	0.12997	0.03212	(0.01549, 1.99875)	0.937	0.07283
	*κ*_2_	0.52466	0.06617	0.02664	(0.06971, 1.12761)	0.929	0.24828

**Fig 10:**
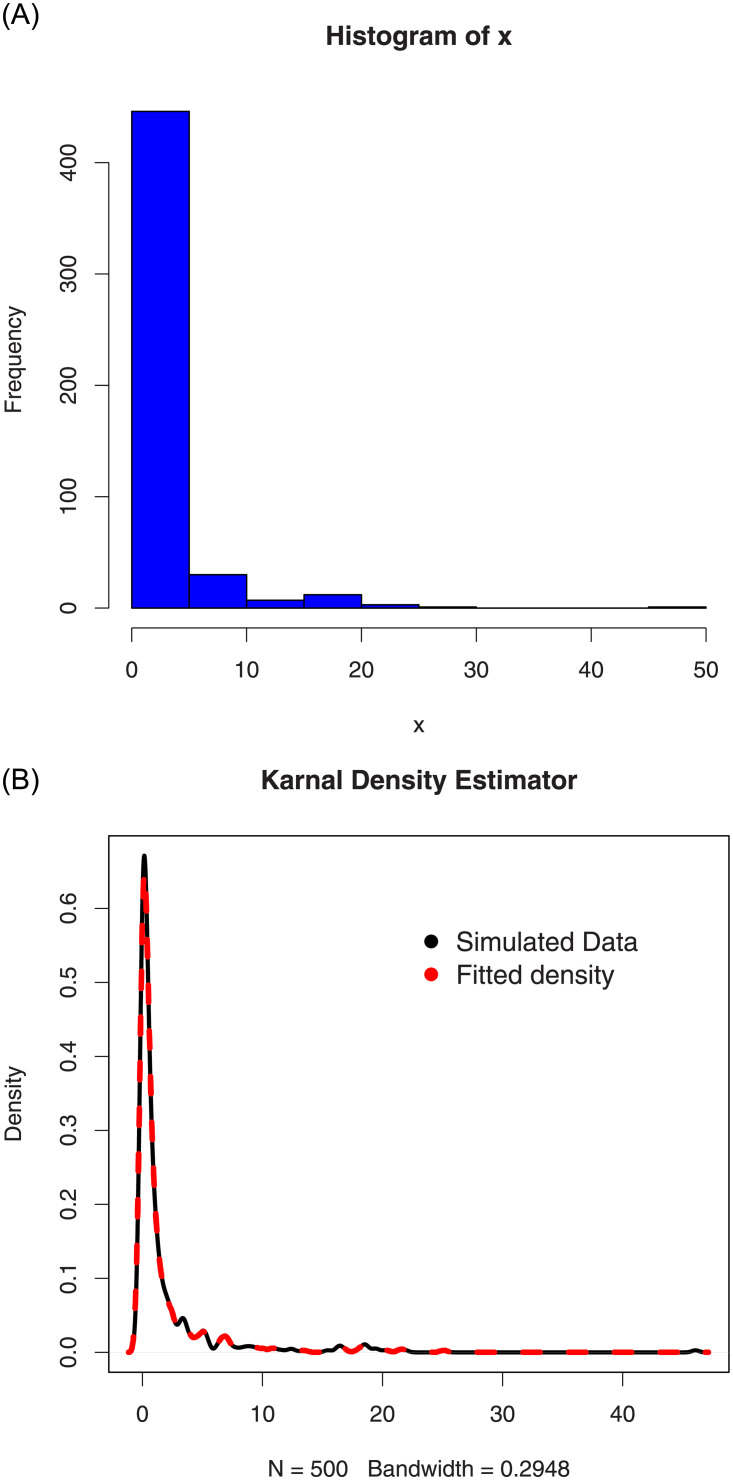
The histogram and Kernel density estimator of the simulated data set 2.

**Fig 11:**
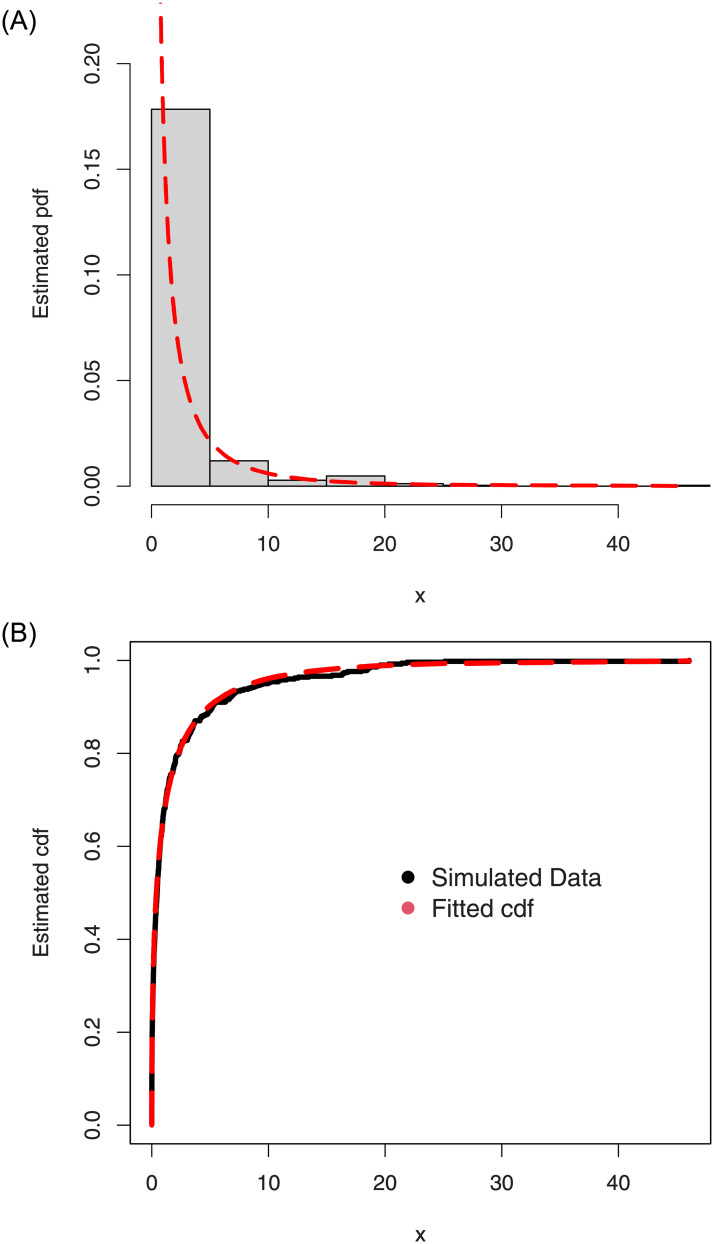
The fitted pdf and cdf of the ASM-Weibull model for the simulated data set 2.

**Fig 12:**
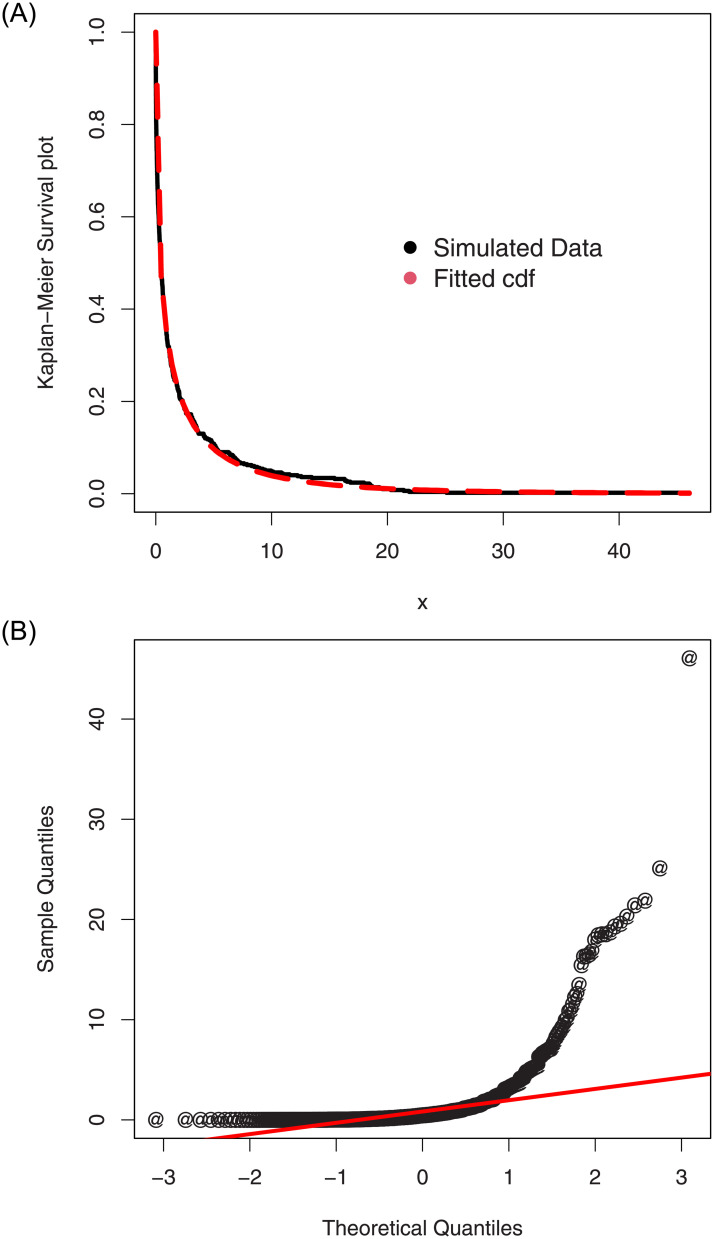
The QQ and Kaplan-Meier survival plots of the ASM-Weibull model for the simulated data set 2.

For the simulated data set 2, (i) the histogram and Kernel density estimator are presented in Fig 10, (ii) the fitted pdf and cdf are sketched in Fig 11, and (iii) the Kaplan-Meier survival and QQ (quantile-quantile) plots are provided in Fig 12. Whereas, the corresponding simulation results are given in Table 3.

**Table 3:** The Monte Carlo simulation results of the ASM-Weibull distribution.

	Set 1: *α* = 0.5, *κ*_1_ = 1.2, *κ*_2_ = 1						
*n*	Par.	MLE	MSEs	Biases	Confidence Interval	CPs	Variances
10	*α*	0.66582	0.53651	0.48321	(0.21252, 0.95390)	0.980	0.13576
	*κ*_1_	1.40571	1.36161	0.50571	(0.74426, 2.15565)	0.932	1.37804
	*κ*_2_	1.38516	0.56682	0.38516	(0.47090, 2.24122)	0.986	1.13841
20	*α*	0.63265	0.47684	0.43265	(0.28860, 0.77669)	0.958	0.10550
	*κ*_1_	1.38657	1.25844	0.46576	(0.69955, 1.83109)	0.912	1.26905
	*κ*_2_	1.31583	0.96998	0.35837	(0.20597, 1.98737)	0.958	1.06494
100	*α*	0.61496	0.43314	0.38556	(0.36773, 0.72455)	0.926	0.09422
	*κ*_1_	1.32931	1.21851	0.40310	(0.40705, 2.39326)	0.910	1.17722
	*κ*_2_	1.27044	0.94830	0.27044	(0.13789, 2.89926)	0.978	1.10603
200	*α*	0.58326	0.37053	0.31410	(0.39251, 0.69401)	0.912	0.05642
	*κ*_1_	1.30819	1.09100	0.35819	(-0.80415, 2.00575)	0.924	1.09821
	*κ*_2_	1.21057	0.76538	0.21057	(-1.2665r, 2.17690)	0.872	1.01865
300	*α*	0.56773	0.27831	0.12262	(0.40840, 0.65870)	0.918	0.02077
	*κ*_1_	1.27689	0.97988	0.16786	(-0.70062, 1.96376)	0.908	0.92467
	*κ*_2_	1.16786	0.71312	0.20786	(-0.78556, 2.10588)	0.900	0.83218
400	*α*	0.53587	0.10437	0.04126	(0.40902, 0.58272)	0.904	0.00996
	*κ*_1_	1.23966	0.68606	0.10673	(-0.53937, 1.67285)	0.920	0.59047
	*κ*_2_	1.10152	0.79517	0.14525	(-0.53139, 1.96108)	0.896	0.68642
500	*α*	0.51290	0.00171	0.00706	(0.42042, 0.56538)	0.966	0.00136
	*κ*_1_	1.21124	0.39390	0.04124	(-0.31099, 1.43478)	0.978	0.47598
	*κ*_2_	1.08239	0.47754	0.09392	(-0.27358, 1.86431)	0.976	0.56431

## 6 Applications to COVID-19 data sets

The main interest of the derivation of the ASM-Weibull distribution is its use in data analysis objectives, which makes it useful in many fields, particularly, in the fields dealing with lifetime analysis. Here, this feature is illustrated via taking two sets of data related to COVID-19 epidemic events.

We illustrate the best fitting power of the ASM-Weibull as compared with the other two parameters, three parameters and four parameters well-known lifetime competitive distributions namely: inverse Weibull (IW), extend odd Weibull exponential (ETOWE), Kumaraswamy Weibull (Ku-W), odd log-logistic modified Weibull (OLL-MW), and Frechet Weibull (FW) distributions. The pdfs of the competitive models are

- Ku-W distribution
  $$\begin{array}{c} f(x)=ab\alpha \kappa_{1}x^{\alpha -1}e^{-\kappa_{1}x^{\alpha }}{\left(1-e^{-\kappa_{1}x^{\alpha }}\right)}^{a-1}{\left[1-{\left(1-e^{-\kappa_{1}x^{\alpha }}\right)}^{a}\right]}^{b-1},\;\;\;\;\;\;\;\;x>0. \end{array}$$
- OLL-MW
  $$\begin{array}{c} f(x)=\frac{a\left(\alpha \kappa_{1}x^{\alpha -1}+\kappa_{2}\right)e^{-a\kappa_{1}x^{\alpha }-a\kappa_{2}x}{\left(1-e^{-\kappa_{1}x^{\alpha }-\kappa_{2}x}\right)}^{a-1}}{{\left(e^{-a\kappa_{1}x^{\alpha }-a\kappa_{2}x}+{\left(1-e^{-\kappa_{1}x^{\alpha }-\kappa_{2}x}\right)}^{a}\right)}^{2}}\;\;\;\;\;\;\;\;x>0. \end{array}$$
- FW
  $$\begin{array}{c} f(x)=\frac{a\alpha b^{\alpha }{\left(\frac{\kappa_{1}}{x}\right)}^{a\alpha }e^{-b^{\alpha }{\left(\frac{\kappa_{1}}{x}\right)}^{a\alpha }}}{x},\;\;\;\;\;\;\;\;x>0. \end{array}$$
- IW
  $$\begin{array}{c} f(x)=ba^{b}x^{-b-1}e^{-{\left(\frac{a}{x}\right)}^{b}},\;\;\;\;\;\;\;\;x>0. \end{array}$$
- ETOWE
  $$\begin{array}{c} f(x)=\alpha \lambda e^{\lambda x}{\left(e^{\lambda x}-1\right)}^{\alpha -1}{\left(b{\left(e^{\lambda x}-1\right)}^{\alpha }+1\right)}^{-\frac{b+1}{b}},\;\;\;\;\;\;\;\;x>0. \end{array}$$

We show that the ASM-Weibull distribution provides the best fit to the lifetime data related to the COVID-19 epidemic. The term “best fit” is used in the sense that the proposed model has smaller values of the criterion selected for comparison. These criterion consist of some discrimination measures. These measures are

- The AIC (Akaike information criterion)
  $$\begin{array}{c} AIC=2k-2\ell , \end{array}$$
- The CAIC (Corrected Akaike information criterion)
  $$\begin{array}{c} CAIC=\frac{2nk}{n-k-1}-2\ell , \end{array}$$
- The BIC (Bayesian information criterion)
  $$\begin{array}{c} BIC=k\log(n)-2\ell , \end{array}$$
- The HQIC (Hannan-Quinn information criterion)
  $$\begin{array}{c} HQIC=2k\log(\log(n))-2\ell , \end{array}$$
  where ℓ is the value of the log-likelihood function under the MLE, *k* refers to the number of parameters of the model, and *n* is the sample size.

In addition to these measures, we also consider other important goodness of fit measures including the Anderson-Darling (AD) statistic, Cramer-von Mises (CM) statistic and the Kolmogorov-Smirnov (KS) statistic with p-value, for detail information about these measures see [35]. A model with the lowest values of the above mentioned measures could be chosen as the best model for the real data set.

For the computation of the numerical results, we use the Newton-Raphson iteration procedure with optim() R-function with the argument method =“BFGS” to estimate the model parameters. The numerical estimates of the unknown parameters of the ASM-Weibull and other fitted distributions are obtained using the R-script AdequacyModel with the “BFGS” algorithm.

### 6.1 Survival times of the COVID-19 patients data

In this subsection, we consider the survival times of patients suffering from the COVID-19 epidemic in China. The considered data set representing the survival times of patients from the time admitted to the hospital until death. Among them, a group of fifty-three (53) COVID-19 patients were found in critical condition in hospital from January to February 2020.

Among them, 37 patients (70%) were men and 16 women (30%). 40 patients (75%) were diagnosed with chronic diseases, especially including high blood pressure, and diabetes. 47 patients (88%) had common clinical symptoms of the flu, 42 patients (81%) were coughing, 37 (69%) were short of breath, and 28 patients (53%) had fatigue. 50 (95%) patients had bilateral pneumonia showed by the chest computed tomographic scans. The data set can be retrieved from https://www.worldometers.info/coronavirus/ and is given by: 0.054, 0.064, 0.704, 0.816, 0.235, 0.976, 0.865, 0.364, 0.479, 0.568, 0.352, 0.978, 0.787, 0.976, 0.087, 0.548, 0.796, 0.458, 0.087, 0.437, 0.421, 1.978, 1.756, 2.089, 2.643, 2.869, 3.867, 3.890, 3.543, 3.079, 3.646, 3.348, 4.093, 4.092, 4.190, 4.237, 5.028, 5.083, 6.174, 6.743, 7.274, 7.058, 8.273, 9.324, 10.827, 11.282, 13.324, 14.278, 15.287, 16.978, 17.209, 19.092, 20.083.

**Fig 13:**
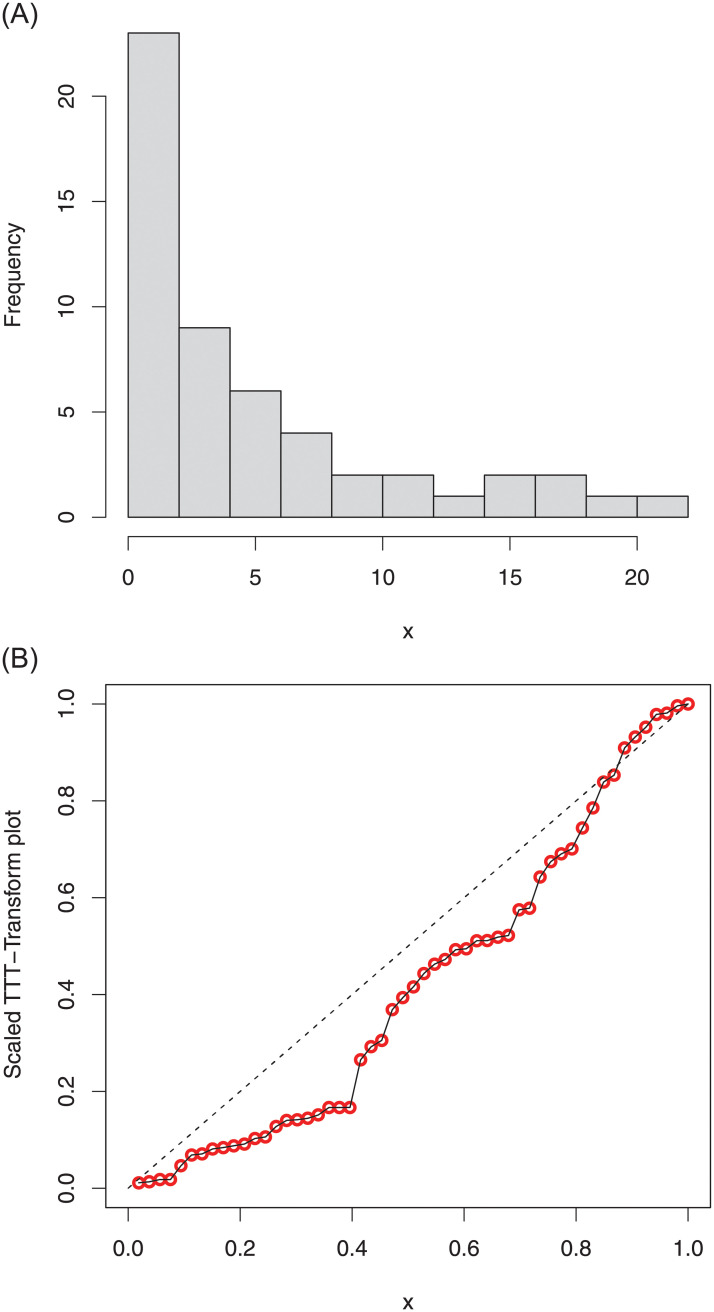
Histogram and TTT plot of the COVID-19 data 1.

The summary measures of the first data are provided in Table 4. Whereas, The histogram of COVID-19 data along with the total time test (TTT) plot are sketched in Fig 13, shows that the data set is right-skewed (histogram).

**Table 4:** The summary measures of the first COVID-19 data.

Min.	1st Qu.	Median	Mean	3rd Qu.	Max.
0.054	0.704	3.079	4.787	6.743	20.083

The MLEs of the ASM-Weibull and other models are provided in Table 5. The discrimination measures of the fitted distributions are provided in Table 6, and the goodness of fit measures are provided in Table 7.

**Table 5:** Corresponding to the first COVID-19 data, the estimated values of the parameters of the fitted distributions.

Model	$\hat{\alpha }$	$\hat{\kappa_{1}}$	$\hat{\kappa_{2}}$	$\hat{\sigma }$	$\hat{a}$	$\hat{b}$	$\hat{\lambda }$
ASM-Weibull	0.8290	0.5332	0.0156				
MOW	0.7046	0.4404		1.4888			
Ku-W	0.7968	3.2500			1.0967	0.0959	
T-MW	0.7864	0.2709	0.0227				0.1145
B-MW	0.4432	2.9385	0.0288		2.8855	0.2178	
OLL-MW	0.7846	0.7693	0.0970		1.9086		
FW	0.5196	0.5045			1.1804	1.9838	

**Table 6:** Corresponding to the first COVID-19 data, the discrimination measures of the fitted models.

Model	AIC	CAIC	BIC	HQIC
ASM-Weibull	272.3406	272.8304	278.2515	274.6136
MOW	273.3886	273.8784	279.2994	275.6616
Ku-W	274.1946	275.0279	282.0757	277.2253
T-MW	274.7410	275.5743	282.6222	277.7717
B-MW	274.0107	275.2873	283.8622	277.7991
OLL-MW	275.1094	275.9765	284.4875	278.0921
FW	292.8950	293.7280	300.7760	295.9260

**Table 7:** Corresponding to the first COVID-19 data, the goodness of fit measures of the fitted models.

Model	CM	AD	KS	p-value
ASM-Weibull	0.0723	0.4555	0.1226	0.4022
MOW	0.0772	0.4957	0.1290	0.3405
Ku-W	0.0740	0.4640	0.1386	0.2600
T-MW	0.0756	0.4750	0.1240	0.3885
B-MW	0.0797	0.4976	0.1435	0.3024
OLL-MW	0.0814	0.5209	0.1506	0.2815
FW	0.2473	1.6930	0.1435	0.2250

From the values of the criteria provided in Tables 6 and 7, we see that the ASM-Weibull model is far from the concurrence. Indeed, for the COVID-19 lifetime data, for instance, it satisfies smaller values of the AIC, CAIC, BIC, HQIC, CM, AD, KS and high p-value against the AIC, CAIC, BIC, HQIC, CM, AD, KS and high p-value for the second-best distribution.

**Fig 14:**
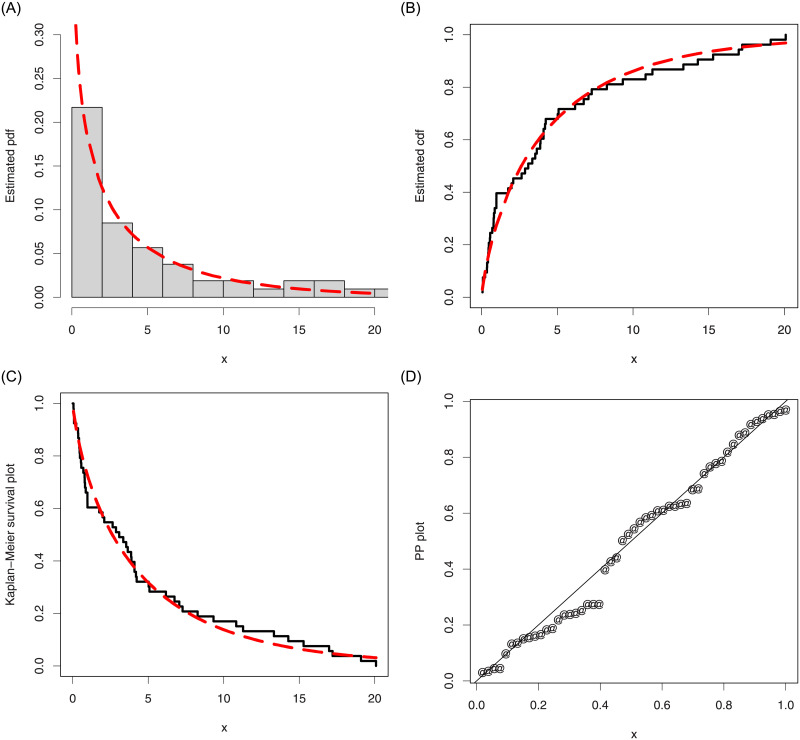
The estimated pdf, cdf, PP and Kaplan-Meier survival plots of the ASM-Weibull distribution for the COVID-19 data.

Furthermore, for the COVID-19 lifetime data, a graphical check of the fit of the ASM-Weibull model are presented in Fig 14. For this purpose, we consider the curves of the estimated pdf, cdf, PP (probability-probability) and Kaplan-Meier survival plots of the ASM-Weibull distribution. For the ASM-Weibull model, the estimated cdf and pdf are given by $G(x;\hat{\alpha },\hat{\kappa_{1}},\hat{\kappa_{2}})$ and $g(x;\hat{\alpha },\hat{\kappa_{1}},\hat{\kappa_{2}})$, respectively, where *G*(*x*;*α*, *κ*_1_, *κ*_2_) is defined by Eq 3, *g*(*x*;*α*, *κ*_1_, *κ*_2_) is defined by Eq 4, and $\left(\hat{\alpha },\hat{\kappa_{1}},\hat{\kappa_{2}}\right)$ are the obtained MLEs for (*α*, *κ*_1_, *κ*_2_). For instance, based on Eq 3, the second row of Table 5, and the plot of the ASM-Weibull distribution in Fig 14 representing the estimated cdf is given by

$$\begin{array}{c} G\left(x\right)=\frac{2}{\pi }arcsine\left(1-e^{-0.5332x^{0.8290}-0.0156x}\right),\;\;\;\;\;\;\;\;\;\;x\geq 0, \end{array}$$

with estimated pdf

$$\begin{array}{c} g\left(x\right)=\frac{2}{\pi }\frac{\left(0.4420x^{0.8290-1}+0.0156\right)e^{-0.5332x^{0.8290}-0.0156x}}{\sqrt{1-{\left(1-e^{-0.5332x^{0.8290}-0.0156x}\right)}^{2}}},\;\;\;\;\;\;\;\;\;\;x>0. \end{array}$$

From the plots sketched in Fig 14, we see that the ASM-Weibull curves are closer to the corresponding empirical objects. The above practical results show that the ASM-Weibull distribution is an efficient model to adjust the considered survival times of the COVID-19’s patients in China.

**Fig 15:**
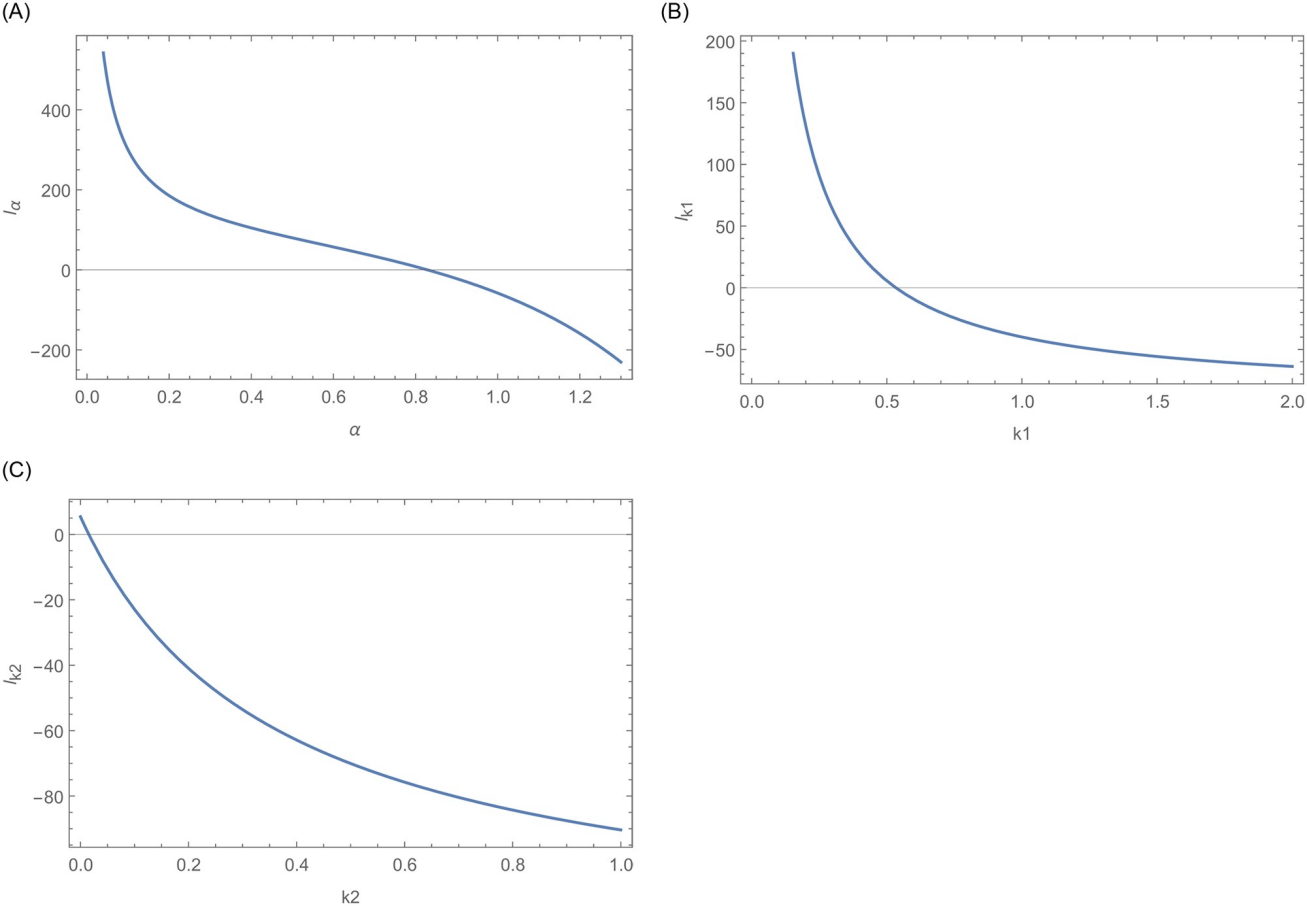
This figure indicates the existences of the log likelihood function as each curve intersect the x-axis at one point.

**Fig 16:**
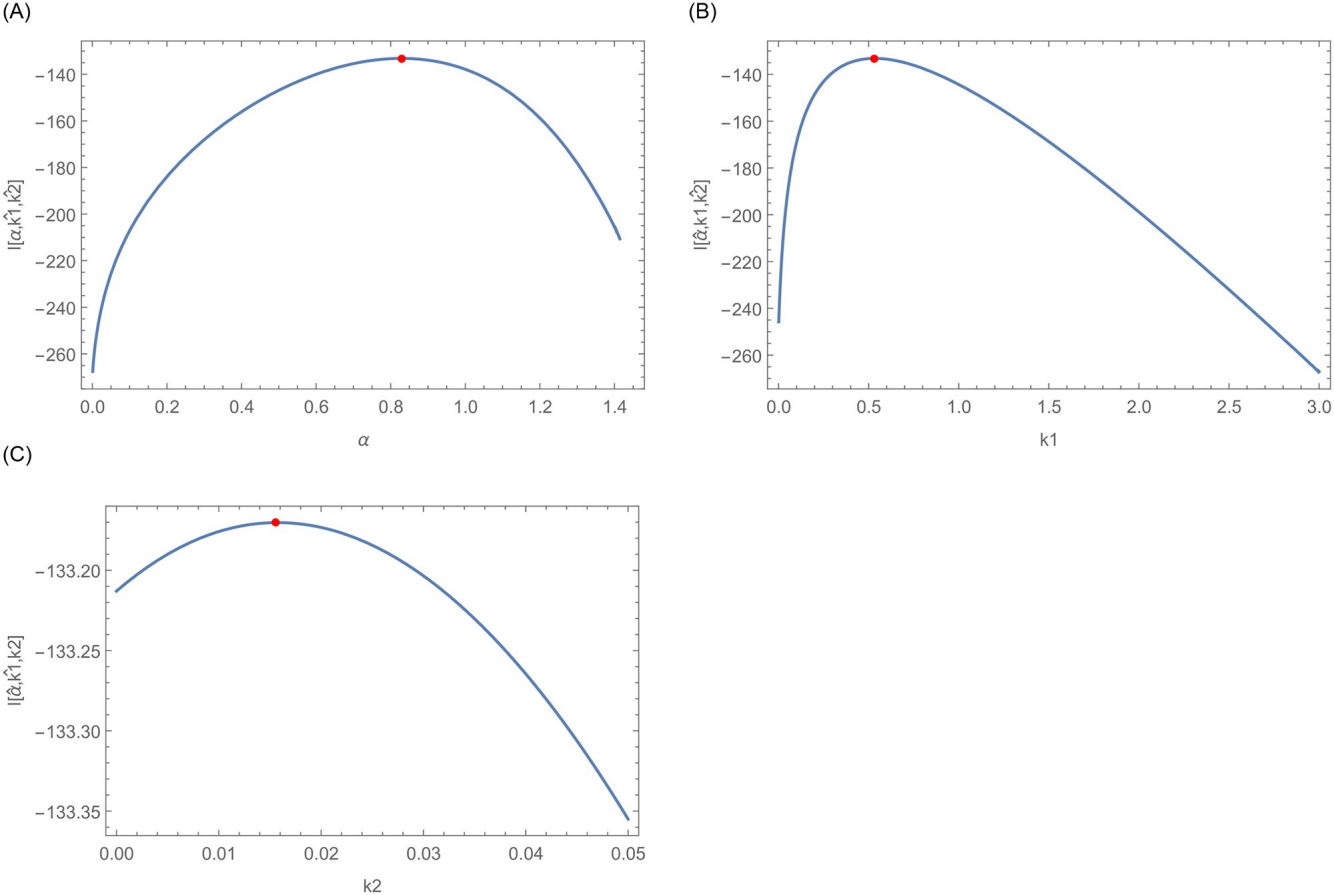
This figure indicates that the log likelihood roots are global maximum.

Furthermore, graphical display of the existence and uniqueness of the MLEs are shown in Figs 15 and 16, respectively. Fig 15 confirms the existence of MLEs as the log-likelihood function intersects the x-axis at one point. Furthermore, Fig 16 shows that the MLEs are unique as the log-likelihood function has global maximum roots.

### 6.2 Second real data set

The second data set represents the mortality rate of the COVID-19 patients in Canada. This data set is available at https://covid19.who.int/, and given by: 3.1091, 3.3825, 3.1444, 3.2135, 2.4946, 3.5146, 4.9274, 3.3769, 6.8686, 3.0914, 4.9378, 3.1091, 3.2823, 3.8594, 4.0480, 4.1685, 3.6426, 3.2110, 2.8636, 3.2218, 2.9078, 3.6346, 2.7957, 4.2781, 4.2202, 1.5157, 2.6029, 3.3592, 2.8349, 3.1348, 2.5261, 1.5806, 2.7704, 2.1901, 2.4141, 1.9048.

**Fig 17:**
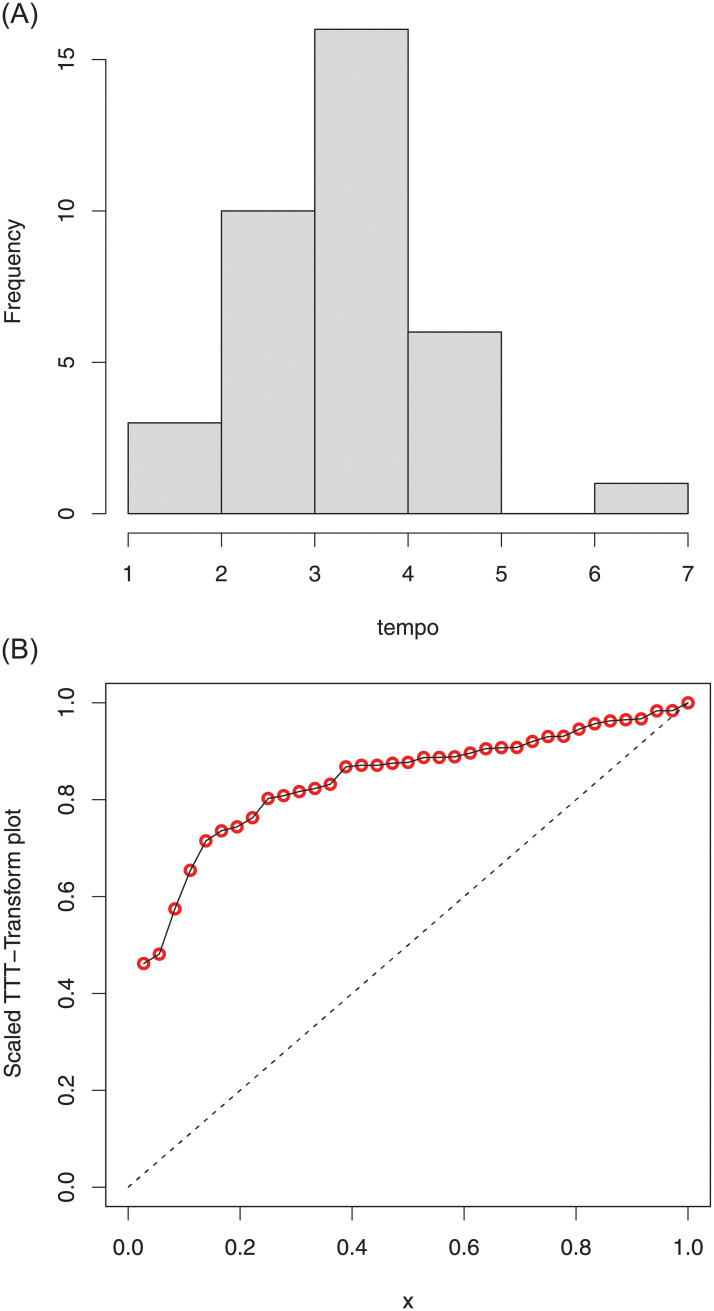
Histogram and TTT plot of the second COVID-19 data set.

Corresponding to this data set, the summary measures are provided in Table 8. Whereas, the histogram and TTT plots are sketched in Fig 17.

**Table 8:** The summary measures of the second COVID-19 data set.

Min.	1st Qu.	Median	Mean	3rd Qu.	Max.
1.516	2.789	3.178	3.282	3.637	6.869

For the second data set, the MLEs of the ASM-Weibull and other models are provided in Table 9. The discrimination and goodness of fit measures are provided in Tables 10 and 11, respectively.

**Table 9:** Corresponding to the second COVID-19 data, the estimated values of the parameters of the fitted distributions.

Model	$\hat{\alpha }$	$\hat{\kappa_{1}}$	$\hat{\kappa_{2}}$	$\hat{a}$	$\hat{b}$	$\hat{\lambda }$
ASM-Weibull	3.47668	0.020029	0.000918			
Ku-W	2.63782	0.448372			1.52414	0.525173
OLL-MW	4.56716	6.99444× 10^−29^	0.001007		0.606009	
FW	1.40346	1.7383			2.25808	2.11702
IW				2.70436	3.16912	
ETOWE	1.07377				5.84409 × 10^8^	1.65812 × 10^8^

**Table 10:** Corresponding to the second COVID-19 data, the discrimination measures of the fitted models.

Model	AIC	CAIC	BIC	HQIC
ASM-Weibull	109.437	110.187	114.188	111.095
Ku-W	119.739	121.03	126.074	121.95
OLL-MW	151.286	152.576	157.62	153.497
FW	113.84	115.13	120.174	116.051
IW	109.84	110.204	113.007	110.946
ETOWE	163.56	164.31	168.31	165.218

**Table 11:** Corresponding to the second COVID-19 data, the goodness of fit measures of the fitted models.

Model	CM	AD	KS	p-value
ASM-Weibull	1.21231	0.212535	0.154695	0.355032
Ku-W	3.52584	0.703955	0.265631	0.0124359
OLL-MW	8.81608	1.96755	0.397571	0.000022
FW	1.61803	0.274479	0.173757	0.227156
IW	1.61803	0.274479	0.173757	0.227156
ETOWE	8.94624	1.86779	0.40961	0.000011

From the values of the selected criteria reported in Tables 10 and 11, we see that the ASM-Weibull model is a better model as it has the smaller values of the AIC, CAIC, BIC, HQIC, CM, AD, KS and high p-value against the AIC, CAIC, BIC, HQIC, CM, AD, KS and high p-value for the second-best distribution.

**Fig 18:**
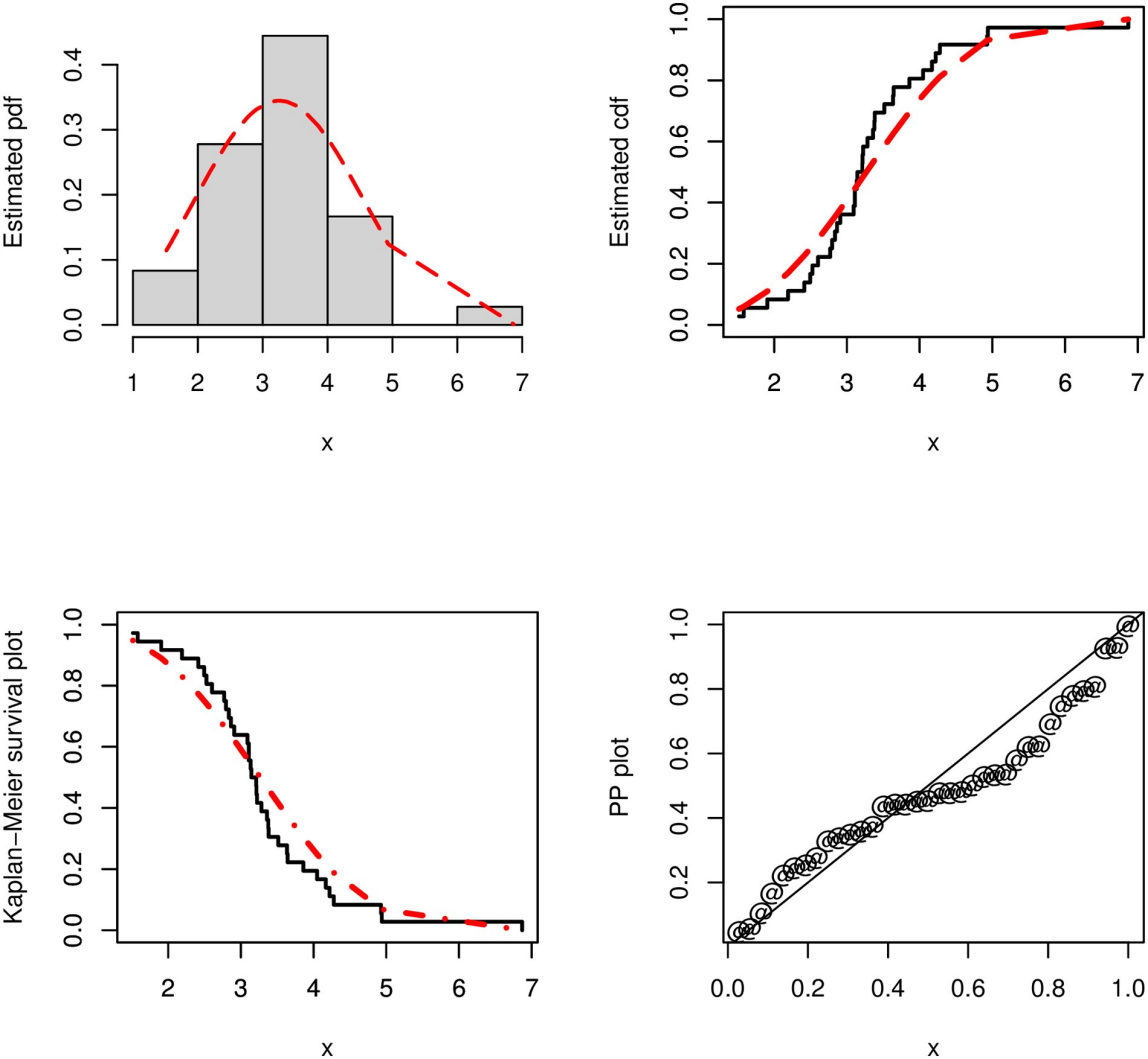
The estimated pdf, cdf, PP and Kaplan-Meier survival plots of the ASM-Weibull distribution for the second COVID-19 data.

**Fig 19:**
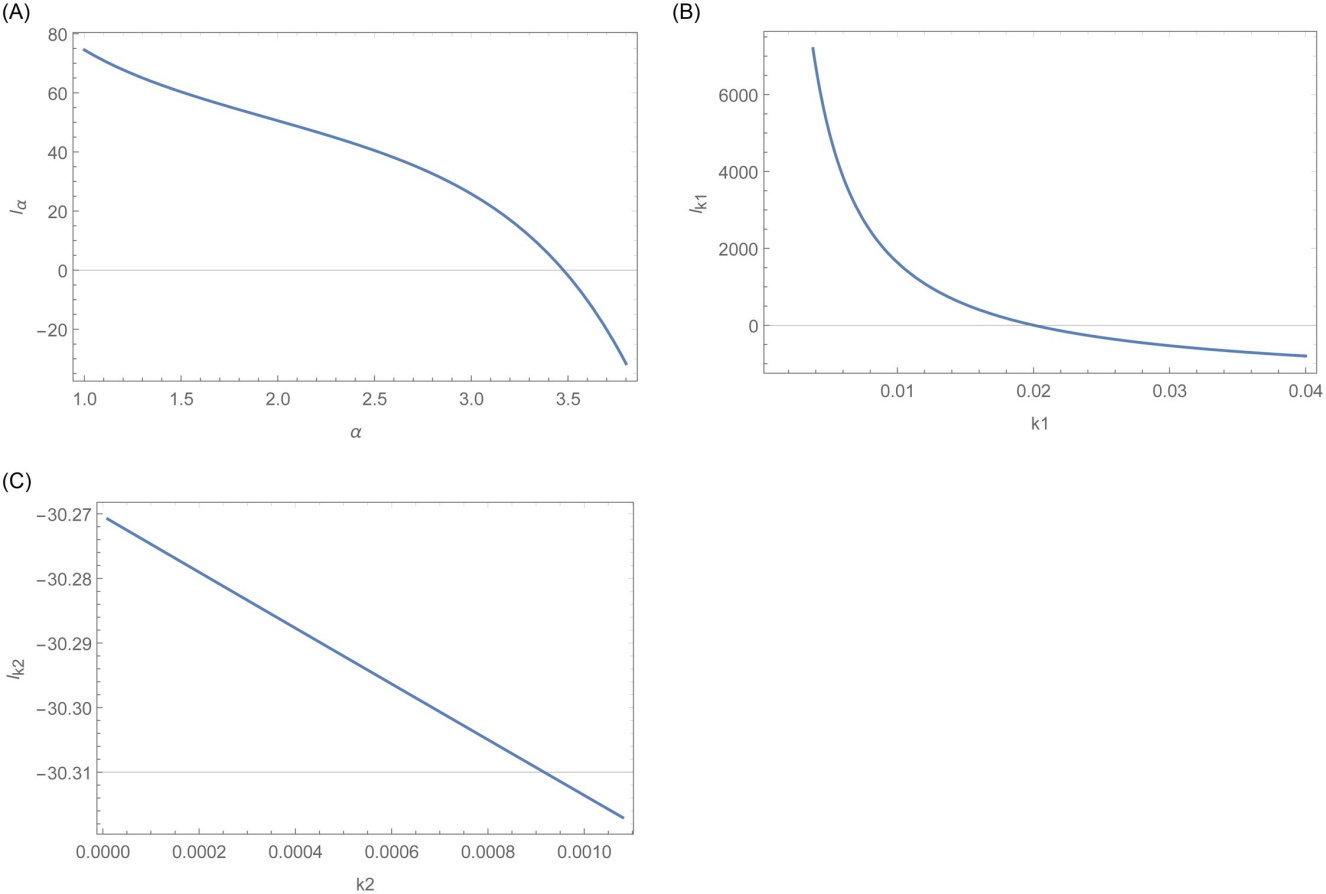
This figure indicates the existences of the log likelihood function as each curve intersect the x-axis at one point for the second data set.

**Fig 20:**
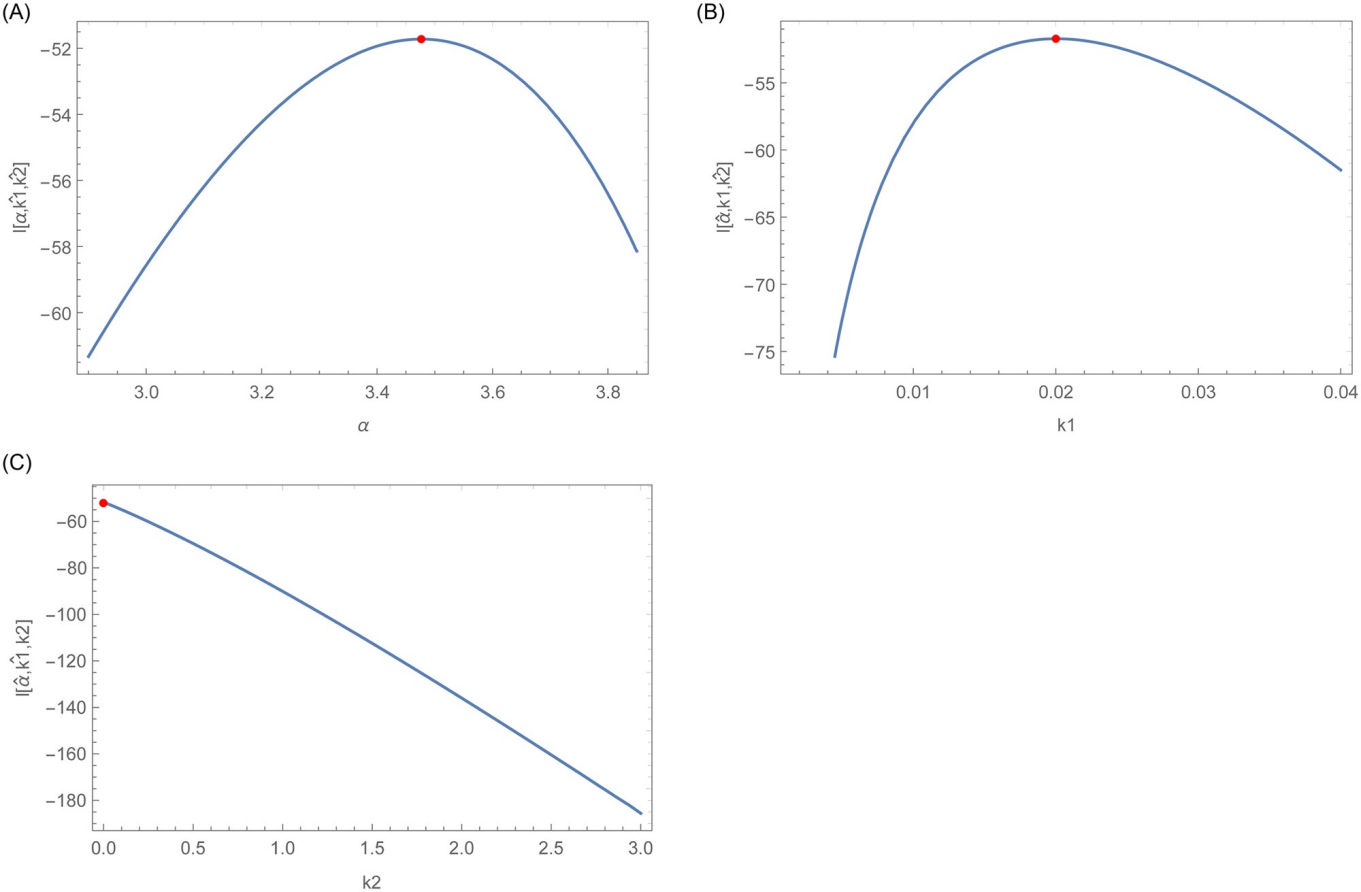
This figure indicates that the log likelihood roots are global maximum for the second data set.

Furthermore, for the second COVID-19 data, the graphical display of the pdf, cdf, PP (probability-probability) and Kaplan-Meier survival plots of the ASM-Weibull distribution are presented in Fig 18. The graphs sketched in Fig 18, show that the ASM-Weibull distribution provide the best description to the COVID-19 mortality rate data. For the second data set, the likelihood function is plotted in Figs 19 and 20, which confirms the existence and uniqueness properties of the MLEs, respectively.

## 7 Concluding remarks

The two-parameter Weibull model has shown great applicability in the practice of statistical sciences particularly, reliability engineering, biomedical and financial sciences. In this study, a new modification of the Weibull model is introduced using the modified Weibull distribution with the “Arcsine strategy”. The proposed model is called the arcsine modified Weibull distribution. The maximum likelihood estimators of the ASM-Weibull parameters are obtained and a Monte Carlo simulation study is conducted. To show the applicability of the ASM-Weibull model, two real-life data sets related to COVID-19 events are considered. The comparison of the proposed model is made with the other well-known competitors. To figure out the close fitting of the fitted distributions, certain analytical tools including four discrimination measures and three goodness of fit measures as well as the p-value are considered. Based on these analytical measures, we showed that the ASM-Weibull model provides a better fit than the other competitors, supported by graphical sketching and numerical tools. Furthermore, corresponding to COVID-19 data sets, the log-likelihood function is also plotted confirming the existence and uniqueness properties of the MLEs. We hope that beyond the scope of this paper, the ASM-Weibull can be applied to analyze other forms of the COVID-19 data.
